# Microglia-associated progression of multiple sclerosis: target identification and therapeutic engagement in human in vitro models

**DOI:** 10.1038/s12276-026-01647-w

**Published:** 2026-02-13

**Authors:** Alica Blenkle, Anastasia Geladaris, Martin S. Weber

**Affiliations:** 1 Fraunhofer Institute for Translational Medicine and Pharmacology ITMP, Translational Neuroinflammation and Automated Microscopy TNM, Göttingen, Germany; 2 Institute of Neuropathology, University Medical Center, Göttingen, Germany; 3 Department of Neurology, University Medical Center, Göttingen, Germany

**Keywords:** Multiple sclerosis, Neuroimmunology

## Abstract

Chronic progression of multiple sclerosis (MS) is likely to develop on the basis of a highly complex interaction of different mechanisms, which are probably already present at disease onset. While animal models have been instrumental in developing therapies for relapsing forms of MS, they have provided limited insight into the processes driving disease progression. To overcome these limitations, human in vitro models have emerged as powerful tools to dissect cellular mechanisms and identify novel therapeutic targets. Here, we highlight advances in modeling MS progression, using human induced pluripotent stem cell-derived systems, with a particular focus on microglia as key mediators of neuroinflammation and neurodegeneration. We critically discuss the strengths and limitations of current induced pluripotent stem cell-based microglia models, and their utility in target identification and therapeutic engagement. By emphasizing translational applications and methodological innovations, this Review provides a framework for leveraging human in vitro models to better understand and therapeutically modulate microglia-associated progression in MS.

## Advancements in iPS cell models transform MS research landscape

Multiple sclerosis (MS) is a disease affecting the central nervous system, leading to inflammation and nerve damage. This Review explores new ways to study MS progression using lab-grown cells. Researchers use in vitro models to mimic human brain cells, including microglia. These models range from simple cell cultures to complex three-dimensional structures called organoids. Organoids can simulate brain environments more accurately, helping scientists understand how MS progresses. The study highlights the potential of using human induced pluripotent stem cells, which can turn into any cell type, including microglia. These induced pluripotent stem cell-derived models offer insights into MS mechanisms and help test new treatments. The findings suggest that, while no model perfectly replicates MS, these advanced techniques provide valuable tools for research.

This summary was initially drafted using artificial intelligence, then revised and fact-checked by the author.

## Introduction

Multiple sclerosis (MS) is one of the most common inflammatory demyelinating diseases of the central nervous system (CNS). Based on clinical observations the disease is classified into relapsing-remitting MS, secondary progressive MS and primary progressive MS^1^. However, this classification has been challenged by evidence of relapses during progressive phases and the early occurrence of progression independent of relapse activity in relapsing MS^2^. While relapse activity is associated with peripheral immune cell activation and CNS infiltration, disease progression is linked to CNS-compartmentalized inflammation^3^. Consequently, it is understandable that modern treatment strategies, such as disease-modifying therapies, which primarily act by modulating the peripheral immune response and are highly successful in targeting relapsing disease activity, fail to prevent disease progression^4^. Thus, developing therapies that effectively target MS progression continues to represent a major unmet need in the field.

Progression of MS is a complex and highly individualized process that is present already at disease onset in all MS types^4,5^. However, understanding and addressing the exact biology underlying progression of MS still remains difficult. Disease progression is probably driven by a combination of several mechanisms rather than a single factor^4,6^. These mechanisms include acute and chronic inflammation, demyelination in both white and gray matter, oxidative stress and mitochondrial dysfunction, which finally induce neuronal loss and neurodegeneration^4,6^. These processes can occur in various combinations either simultaneously or separately and, together with the failure of repair mechanisms such as remyelination, ultimately drive disease progression^7^. Because these processes occur within the CNS, the role of microglia in promoting disease progression has emerged, particularly given their involvement in all stages of MS and in several of the aforementioned pathomechanisms^8,9^.

Microglia are the innate immune cells of the CNS, displaying high functional plasticity and adaptability to environmental and pathological changes^10^. They serve as the first line of immune defense and function in synapse pruning, injury repair, homeostasis maintenance and regulation of brain development through scavenging and phagocytosis. Moreover, they play a pivotal role in neuroinflammation, tissue repair, neural homeostasis and neurodegenerative diseases^10^.

In MS, various microglial phenotypes arise within different lesion types. These activated phenotypes are distinguished by reduced expression of homeostatic markers and increased expression of pro-inflammatory markers^11^. Although microglia generally exhibit a more pathogenic phenotype in MS, the loss of critical homeostatic functions potentially contributes to increased damage and reduced repair, probably driving disease progression^6,9^.

Given their central role and sustained involvement in MS progression, microglia represent an attractive potential therapeutic target. However, to introduce specific treatment strategies, there is an urgent need to gain a better understanding of the underlying mechanisms, which promote disease progression. One major obstacle is the limitation by a paucity of relevant preclinical animal models^12^. Animal models have been useful in the development of therapies targeting relapse disease activity, but have been largely unsuccessful in the identification of therapies that are halting and/or reversing MS progression, as they reflect only some aspects of the disease. Moreover, the models lack essential human characteristics and are associated with numerous species-species differences^12^. The lack of adequate animal models that mimic MS progression has driven efforts to develop new technologies based on various in vitro tools. Starting from human-derived primary cell cultures to induced pluripotent stem (iPS) cells in both two-dimensional (2D) and complex three-dimensional (3D) cell culture systems. Although none of these methods can fully capture the complexity of MS, they show promising results in terms of their capacity to recapitulate certain mechanisms.

In this Review, we will aim to discuss the advantaged and disadvantages of current available in vitro models in reflecting and understanding microglia-associated disease progression and in their potential to develop future effective therapeutic strategies for MS progression.

## In vitro models in MS

**Fig. 1:**
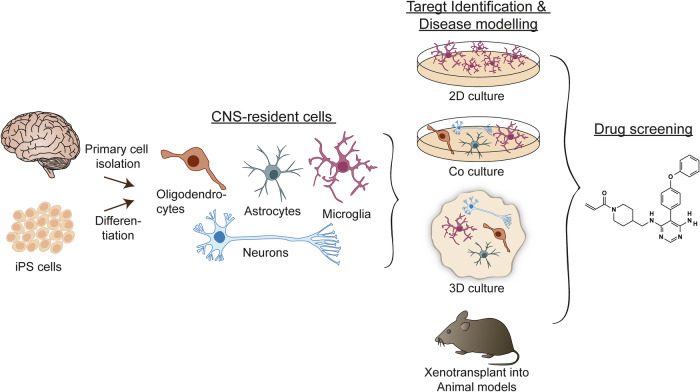
Schematic representation of the potential applications of primary cells and iPS cell-derived in vitro cell models. CNS-resident cells can be isolated from human tissue or generated by differentiation of iPS cells to specific cell types. The cells can be cultured in 2D cultures, coculture systems or 3D cultures or directly transplanted into animal models of MS. Ultimately, they serve as a platform for disease modeling and drug screening, which can facilitate the development of novel therapies for MS.

Many in vitro models have been generated to mimic microglia phenotypes and functions in health and disease^13–15^. Furthermore, in vitro studies allow both in-depth and high-throughput analysis. The complexity of available models varies from simple monolayers obtained from primary cultures or immortalized cell lines to more complex multicellular 3D models, which we will discuss in the following section and which are summarized in Fig. 1 and Table 1.

**Table 1:** Summary of the benefits and limitations of current in vitro models.

Cellular models	Advantages	Limitations/challenges
Primary murine cells	Preservation of main cellular features/Genetically modifiable	Loss of brain environment dependent signatures/Loss of functions dependent on cell–cell interactions/Typically, do not fully recapitulate adult signatures/Cannot fully represent human genetics/Low throughput
Primary human cells	Preservation of main cellular features/MS-relevant pathways preserved	Loss of brain environment dependent signatures/Loss of functions dependent on cell–cell interactions/Low throughput/Ethical concerns
iPS cell monoculture	Scalability/High-throughput screens possible/Easily genetically modifiable/Use of patient-derived cell lines possible	Loss of brain environment dependent signatures/Loss of functions dependent on cell–cell interactions/Not all differentiation protocols are robustly reproducible
iPS cell 2D coculture	Improved microglia maturation/Investigation of microglia interactions with other CNS-resident cells possible/Genetics of each individual cell type can be modulated	Transcriptome more closely resembles fetal than adult microglia/Not all CNS cell types are modeled/Lack of viscoelastic environment present in the brain
iPS cell 3D coculture	Microglia embedded in CNS environment and can interact with various cell types/Transcriptome resembles in vivo microglia/Robust protocols published/Genetics of each individual cell type can be modulated in spheroids/Models with BBB and vascularization available	Long generation and maturation times/Depending on protocol used, transcriptome more closely resembles fetal or adult microglia/Low throughput/Heterogeneous cell composition and differentiation in organoids/Inconsistent reproducibility/If not vascularized, necrotic core formation
Xenotransplantation	Microglia mature within physiological brain environment/Transcriptome resembles adult in vivo microglia/Microglia genetics can be manipulated/Transplantation into animal disease models possible/Physiological BBB present/Vascularization	Long generation and maturation times/Low throughput/Different genetic background between host and engraftment/Variable engraftment efficiency/Host microglia present unless pharmacologically or genetically eliminated

### Primary murine cell cultures

Primary microglia isolated from rodent brains (isolation and culture methods have been reviewed elsewhere^16^) are widely used to examine the direct effects of potential therapeutics targeting microglia-associated MS progression and their possible mechanisms of action. For instance, studies in primary murine microglia suggested that a dysregulation of adipokines increases the risk of developing MS and plays a role in its pathology^17,18^. This hypothesis was further investigated in patients with MS, where lower plasma and brain expression levels of the anti-inflammatory adipokine apelin were found. As a result of these findings, apelin was supplemented in models of neurodegenerative diseases as a therapeutic strategy, which revealed neuroprotective and anti-inflammatory effects^18–20^. As an alternative to apelin, a more plasma-stable apelin peptide (LIT-01-200) additionally demonstrated neuroprotective effects and regulation of microglia inflammatory responses in the MS animal model experimental autoimmune encephalomyelitis^20^. These findings were supplemented with in vitro studies, showing that LIT-01-200 reduced the pro-inflammatory state of primary murine microglia.

Another strategy to control MS progression that has gained interest is the inhibition of the enzyme Bruton’s tyrosine kinase (BTK). BTK plays a role in the activation of B cells and myeloid cells, such as microglia, and its expression is increased in active and chronic active lesions of patients with MS^21–24^. In various animal models of MS, BTK inhibition reduced microglia-mediated inflammation and promoted remyelination^23^. To correlate the observed phenotypic changes with functional changes in microglia, BTK inhibition was evaluated in primary murine microglia. These investigations showed that inhibition of BTK reduced pro-inflammatory activation of microglia and enhanced their phagocytosis capacity^23^.

These studies demonstrate the usefulness of primary rodent microglia in investigating microglia phenotypes. However, their culture time is limited, and the isolation and culture process cause changes in their gene and protein expression, as well as their phenotype, as microglia function and identity are highly influenced by their microenvironment^25–30^. This is reflected by a lower expression of homeostatic genes, a higher immune activation and an increased expression of axon tract-associated microglia genes^30–32^. Furthermore, rodent primary microglia are typically isolated from neonatal brains. Because microglia undergo substantial changes and maturation processes after birth, these cells might not fully recapitulate adult microglia owing to their retention of an immature phenotype. In addition, rodent primary microglia cannot fully represent human genetics. Studies of multigenic diseases have shown that, between mice and humans, the homology of disease-associated genes is low, and the expression levels of complement system genes, various inflammatory cytokines and genes related to neurodegenerative diseases differ^30,33–35^. These species-to-species differences reduce the reliability of rodent models to reflect human disease pathology, including MS, obstructing the translation of preclinical research^36^. Therefore, findings from rodent cells require validation in human model systems.

### Primary human cell cultures

To overcome the described species-to-species differences, primary human microglia can be derived from post-mortem tissue or perioperative brain resections by isolation via fluorescence-activated cell sorting, immune-panning, magnetic-activated cell sorting or percoll gradient centrifugation^35,37–41^. As with rodent primary microglia, separating human microglia from their in vivo environment causes substantial changes in gene expression, phenotype and function^30,42,43^.

Research on primary human microglia is limited by the scarce availability of human brain tissue, low yields after isolation, lack of proliferation, variability in culture quality, and ethical concerns, highlighting the need for alternative human models^44^.

Nevertheless, some studies have used primary human microglia to investigate microglia functions in the context of MS. For instance, primary human cells were used to examine whether changes in myelin from the normal-appearing white matter of patients with MS influence its phagocytosis by microglia^45^. Independent of the oxidation status of myelin, myelin uptake was more efficient from donors with MS compared with healthy control donors. In addition, it was observed that, with increasing age of the myelin donors, myelin phagocytosis was reduced, whereas myelin phagocytosis increased with microglia donor age^45^. These findings suggest that, in MS, changes in myelin promote its phagocytosis by microglia, and that while the susceptibility of myelin to phagocytosis decreases with age, the phagocytic capacity of microglia is enhanced.

### iPS cell cultures

To overcome the scarcity of primary human cells, human iPS cells have emerged as a potential reproducible model more closely connected to human pathology. iPS cells can be differentiated into simple monoculture or more complex 3D organoids, which can be utilized as a model to explore more intricate cellular interactions^13,46^. Similar to primary human cells, human iPS cell-derived cell cultures offer the advantages of a human genetic background, which improves the recapitulation of disease-associated gene orthologs, with the additional benefit that iPS cell-derived cultures enable the availability of large cell amounts^47^. Therefore, they hold the potential to enhance the success rate of translating preclinical studies into the clinic, as well as allowing high-throughput analysis. Further advantages are the suitability of iPS cells for gene editing to investigate disease-relevant gene variations, and they allow the generation of patient-derived cells, especially for polygenetic diseases.

The introduction of embryonic stem (ES) cells and iPS cell technology has led to the generation of CNS-resident cells derived from the neuroectodermal lineage^48,49^. As microglia are of hematopoietic ontogeny, their generation proved to be more difficult^50^. However, advances in the field have led to the development of various iPS cell microglia differentiation protocols that have been comprehensively discussed and reviewed elsewhere^34,46,51^.

In general, the protocols mimic the main characteristics of primitive hematopoiesis, including dependence on the transcription factors PU.1 and IRF8, independence from MYP, and reliance on WNT signaling^52–57^. A large proportion of the differentiation strategies accomplish the initial myeloid differentiation by the use of growth factor and cytokine cocktails. Then, the maturation into microglia requires CNS environmental cues, which is accomplished by various final medium compositions and the use of monoculture or coculture with other CNS-resident cells^34,54,58–73^. Moreover, protocols have been established that rapidly and effectively induce the generation of microglia via the expression of a combination of transcription factors^74–78^.

The generated human iPS cell microglia were shown to recapitulate microglial states identified in adult human brain microglia in response to CNS-derived stimuli, allowing the investigation of microglial phenotypes in homeostatic and disease-associated contexts^79^. Furthermore, a recent study revealed that iPS cell microglia derived from patients with MS (MS microglia) possess intrinsic alterations in their basal state compared with microglia from healthy donors (HCs)^80^. In particular, MS microglia displayed a decreased expression of the homeostatic microglial gene *P2RY12*, which is in accordance with the loss of expression of microglial *P2RY12* in brain autopsies from patients with MS^81,82^. In addition, MS microglia displayed a specific transcriptional signature with changes in genes linked to MS pathology and microglial states in MS, as well as an upregulation of the recently identified immune-related transcripts PIGR, BST1 and FPR2 and the long noncoding RNA XIST linked to female sex-biased autoimmunity and regulation of microglia and macrophage responses^80,83–85^. The dysregulation of BST1 and XIST remained following lipopolysaccharide stimulation, indicating a cell-autonomous inflammatory state resistant to acute inflammatory stimulation^80^. Moreover, genes involved in immune regulation and oxidative stress were upregulated in MS versus HC microglia, whereas genes related to extracellular matrix organization, cell adhesion, cell shape regulation and wound healing were downregulated. While the study revealed few changes in the secretion of inflammatory cytokines, the internalization of zymosan beads was increased in MS compared with HC microglia, indicating that alterations in phagocytosis ability play a role in MS pathology. However, as the authors discuss, elucidating the mechanisms involved in dysregulated phagocytosis and clearance of myelin in MS requires further studies using myelin debris^80^. Overall, this study demonstrated the usefulness of iPS cell microglia derived from patients with MS to investigate microglia reactivity in MS.

While iPS cells from HCs and patients with MS are a useful tool to study microglia phenotypes and functions, reprogramming of somatic cells into iPS cells requires various changes in epigenetic modifications to obtain pluripotency, which can affect the final differentiated microglia^86^.

Incomplete epigenetic reprogramming can result in residual epigenetic modifications, often termed epigenetic memory, as well as de novo epigenetic changes induced during reprogramming. Such alterations can lead to variability in genomic stability, tumorigenic potential, differentiation capacity and the expression of disease-relevant genes across iPS cell lines^87–97^. While the difference in differentiation potential into a specific cell type can be harnessed by selecting an iPS cell source that matches the desired differentiated cell type or be reduced by extended iPS cell passaging, differences in gene expression can lead to an over- or underestimation of the phenotype presented in patient-derived cells^91,98–101^. However, donor-specific epigenetic memory could also be used to study epigenetic mechanisms that play a role in MS progression, which requires in-depth epigenetic profiling of iPS cell donors, the reprogrammed iPS cells and differentiated cells^102–106^. Notably, iPS cell microglia more closely resemble the transcription profile of primary fetal microglia than adult cells, which could impact the investigation of age-related processes associated with diseases such as MS^61^.

### Cocultures

The previously discussed microglia monoculture models cannot fully recapitulate human in vivo microglia owing to the lack of CNS environmental cues, which causes substantial changes in gene expression and does not fully represent homeostatic gene signatures^15,28,30,42^. Furthermore, microglial states, as well as their response to stimuli, are shaped by the interaction with other CNS-resident cells and vasculature^107,108^.

To investigate not only cellular interactions mediated by soluble factors but also by physical contact, coculture systems with other CNS-resident cells are required. Of note, with increasing complexity and physiological relevance of the culture system comes a decrease in experimental control^46^.

In addition, the microglial homeostatic, more quiescent state is maintained through receptor–ligand interactions, such as neuronal CD200 with the microglial CD200 receptor and neuron membrane-bound CX3CL1 with microglial CX3CR1 receptors, further supporting the need for coculture models^109–112^.

Coculturing microglia with other CNS cell types provides some CNS environmental cues and is able to partly rescue the microglial phenotypes lost in monoculture. For instance, coculture of hematopoietic progenitors or macrophages with astrocytes or neurons facilitates microglia differentiation, promotes their maturation and improves ramified morphology and motility^54,58,61,65,67^. In addition, coculture systems allow the investigation of cellular interactions and the relative contributions of the different cell types.

Cellular cross-talk of microglia and other CNS-resident cells is essential in the pathophysiology and resolution of MS^6,113–115^. Therefore, a combination of monoculture and coculture models derived from primary cells or iPS cells allows mechanistic studies and the dissection of cell-type-specific contributions in a disease-relevant model. While cocultures of primary human or rodent microglia with other CNS-resident cells are technically possible, they are not routinely used in research on neurological disorders, where most studies have established iPS cell-based coculture models^22,61,68,70,116–120^. For example, in several iPS cell-based models of Alzheimer’s disease, coculture of microglia, astrocytes and/or neurons has provided insights into disease-related cellular cross-talk^61,68,70^. Moreover, a recent study investigating the relevance of BTK in MS utilized iPS cell microglia and a triculture system consisting of neurons, astrocytes and microglia to evaluate the role of BTK in microglia^22^. Using these models, it was shown that the BTK-dependent effects observed in triculture were mainly driven by microglia whose inflammatory response was mediated by BTK-dependent signaling.

Although these studies demonstrated that 2D coculture models are a useful tool to decipher microglia function and interaction with other CNS cell types, these systems cannot completely recapitulate the complex brain environment.

### iPS cell 3D cultures

In the CNS, microglia are embedded in a complex network of neuronal and glial cells, as well as macromolecules produced by these cells, resulting in a viscoelastic environment that shapes the morphology and phenotype of microglia^121^. Culture conditions in monoculture and 2D coculture largely lack these properties leading to in-vitro-specific changes in phenotype and gene expression^28,30,42^. To represent a more homeostatic phenotype in iPS cell microglia, 3D culture models provide an extracellular environment more closely resembling the in vivo conditions.

Several strategies exist to incorporate microglia into a more physiologically relevant environment, such as migration and engraftment of separately derived microglia into brain spheroids and organoids (assembloids)^58,63,66,122–124^.

#### Spheroids

The least complex 3D culture model in which iPS cell microglia can be integrated is spheroids. Spheroids are cellular systems derived from separately patterned brain cell lineages that display limited self-organization and can be utilized to investigate particular CNS cell types in a 3D environment with high consistency in cellular composition^125^. Microglia embedded in neuron/astrocyte spheroids display highly ramified projections and are able to respond to injury^63^.

Spheroids allow the investigation of CNS cell interactions in a simplified and more closely controlled environment. The more homologous nature of the model increases reproducibility, making spheroids suitable for drug screens. Furthermore, brain spheroids containing microglia and a functional blood–brain barrier (BBB) have been generated^126,127^. Compounds targeting MS progression are required to pass the BBB and modulate cell types within the CNS. Hence, such spheroid models could serve as tools to study BBB penetrance of novel therapeutics and their modulatory effect on CNS-resident cells, including microglia, in a single model during drug discovery.

#### Brain organoids and assembloids

While spheroids are simplified models lacking cortical organization, brain organoids more closely mimic brain tissue architecture^125^. To examine brain region interactions and neural circuit development, region-specific organoids potentially containing multiple cell lineages are fused to generate assembloids. By incorporating non-neural cell types, such as blood vessels or microglia, into brain region-specific organoids to form multilineage assembloids, neurovascular and neuroimmunological interactions can be assessed^128–130^.

Microglia populating brain organoids present ramified morphologies, are able to respond to injury and pro-inflammatory stimuli, and phagocytose Aβ^58,66,122,124^. Moreover, microglia engrafted into ventral and dorsal forebrain organoids display different phenotypes, which demonstrates the ability to depict region-specific diversity of microglia in vitro^124^.

During brain organoid generation, signaling pathways essential for mesoderm formation are commonly inhibited to quickly induce neuroectoderm formation, thus preventing microglia production within the organoid. However, when brain organoids are generated without dual-SMAD (Small Mothers Against Decapentaplegic) inhibition, microglia innately develop within the organoids (organoid-grown microglia, oMG)^131,132^. oMG resemble ex vivo human adult microglia but still cannot fully recapitulate human homeostatic microglia as oMG express homeostatic microglia genes, *TMEM119* and *P2RY12*, at much lower levels than ex vivo human microglia^131^. In addition, the generation of microglia in cortical organoids (microglia-containing human cortical organoids, mhCOs) induced by the overexpression of the transcription factor PU.1 has recently been demonstrated^133^. Based on the comparison of single-cell RNA sequencing data, the transcriptional profile of microglia within mhCOs more closely resembles primary fetal microglia than oMG, which in turn shows a stronger correlation with adult microglia signatures than fetal ones^131,133^. However, a more comprehensive comparison between different iPS cell microglia models, primary cultured microglia and acutely isolated human adult microglia is required to determine which microglia models exhibit a transcription profile more similar to that of human in vivo microglia.

In a recent study, the macroglia–microglia axis that sustains chronic inflammation in MS progression was examined using immunocompetent mature glia-enriched organoids^134^. To create a complex dysregulated inflammatory environment, the organoids were treated with inflamed cerebrospinal fluid (CSF) from patients with MS. In response to CSF exposure, drastic transcriptional changes occurred in microglia. In particular, genes regulating the inflammatory response, antigen processing and presentation, cytokine–cytokine receptor interaction, activation of the Toll-like receptor signaling pathway, complement pathway, reactive oxygen species production, ferroptosis and interferon-γ response were affected. Furthermore, the transcriptional profile of the CSF-treated microglia partially overlapped with the profile of microglia found at the chronic active lesion edge from post-mortem brain tissue of patients with MS^81,134^. In addition, CSF-exposed organoids displayed an increased number of interactions between microglia and astrocytes. The model presented by Fagiani et al. therefore demonstrated that microglia within brain organoids enable the investigation of the microglia transcriptome and the cross-talk between microglia and other CNS-resident cells in a disease-relevant context^134^.

In a following study, Fagiani et al. used the previously established organoid model to investigate cellular senescence, where cells undergo cell cycle arrest and present with a senescence-associated inflammatory phenotype, as part of the mechanisms promoting CNS-compartmentalized inflammation in MS progression^135^. First, an increase in senescent-like cells in the white matter of patients with MS, especially in microglia, astrocytes and epithelia, was identified by transcriptomic analysis of autopsy brain tissue from relatively young patients with progressive MS. Among the lesion pathological stages, the localization of cells with a senescent signature was present particularly at the edges of chronic active lesions, suggesting that inflammation triggers cellular senescence and could be linked to MS lesion pathology. Then, to examine the role of inflammation-induced senescence in MS, organoids generated from iPS cells derived from patients with MS were exposed to 10% CSF from untreated patients with MS that exhibited chronic active lesions, followed by the assessment of senescence markers. The organoids exposed to this CSF presented with increased senescence-like cells, which was additionally observed in organoids exposed to a cocktail of the pro-inflammatory cytokines IFN-γ, TNF-α, IL-1β and, C1q. In accordance with transcriptomic data from autopsy MS brain tissue, microglia and astrocytes in particular displayed cellular senescence, leading to upregulation of cell cycle arrest genes as well as genes related to inflammatory processes, including cytokine signaling and antigen presentation, in senescent-like microglia. These findings indicate that, in MS, inflammatory signals initiate cellular senescence. The authors propose that these processes occur early in MS pathology and promote CNS-compartmentalized inflammation by, for instance, releasing inflammatory mediators and impairing debris clearance by senescent-like microglia. The increased senescence-like processes in MS CSF-treated organoids could be reduced using anti-inflammatory drugs ibudilast (posphodiesterase type 4 and macrophage MIF inhibitor), α-lipoic acid and tolebrutinib (BTK inhibitor), suggesting that targeting inflammatory-driven cellular senescence could attenuate MS progression^135^.

In MS, the role of microglial lipid metabolism has gained importance and is seen as a potential therapeutic target^115,136,137^. In post-mortem brain tissues of patients with MS, lipid-droplet-accumulating or foamy macrophages/microglia have been detected^138–140^. This microglia phenotype has been linked to impaired remyelination and intermediate inflammatory states, indicating the importance of targeting microglial lipid metabolism in mitigating disease progression^136,139,141,142^. Microglia with a dysregulated cholesterol metabolism disrupted oligodendrogenesis in a human neural assembloid model of Aicardi–Goutières syndrome^143^. Goldberg et al. proposed that the mechanism by which the impaired cholesterol metabolism observed in Aicardi–Goutières syndrome-associated microglia disrupts white matter homeostasis is similar to that observed in MS^143^. Therefore, the model might be adapted and/or modified to investigate microglial lipid metabolism and its modulation in MS progression.

Moreover, another myelinating brain organoid model integrating microglia showed that, in response to demyelination induced by lysolecithin, microglia downregulated genes related to cholesterol metabolism that were upregulated during remyelination^144^. In addition, the presence of microglia induced oligodendrocyte precursor proliferation and oligodendrocyte differentiation, indicating that microglia support oligodendrocyte functions and thereby promote remyelination. One way microglia may support oligodendrocyte differentiation during remyelination is by promoting regulatory processes involving retinoic acid, as indicated by the upregulation of the retinoic acid-related gene *RXRA* (retinoic X receptor alpha) in organoids containing microglia. RXR agonism was previously reported to facilitate remyelination and has been investigated for its therapeutic potential in patients with MS^145–148^. Using lysolecithin-induced demyelination in the myelinated organoid model further showed that the pro-remyelinating drug clemastine promotes remyelination not only by supporting oligodendrocyte differentiation but also by reducing microglia-mediated inflammation, highlighting the utility of this model in elucidating the mechanisms of action of therapeutic compounds^144^.

Despite the advances in 3D iPS cell modeling, a technical challenge that still needs to be overcome is the high variability between repeated organoid differentiations. Moreover, spheroid, organoid and assembloid technologies are technically complex and expensive, and still cannot recapitulate a fully mature and structurally organized, vascularized brain environment. As vasculature is required for a consistent supply of oxygen and nutrients within the brain, the lack of vascularization restricts long-term survival and size of 3D models as well as deprives neural stem cells of supporting factors that promote neurogenesis and astrocyte development^149–152^. Together with a limited removal of metabolic waste products, lack of gas exchange and nutrients contributes to the formation of a necrotic core that hampers physiological cell development and migration^153,154^. When studying mechanisms involved in MS progression, these limitations hamper the investigation of chronic inflammatory processes in these models. However, incorporating microglia into brain organoids improves the viability of neuronal populations through removal of cellular and axonal debris^144,155^. In addition, slicing of organoids or culture oscillation can improve cellular viability, and vascularized organoid models have been developed to mitigate necrotic core formation^156–161^.

### Xenotransplantation models

To provide a more mature and structurally developed environment, iPS cell microglia, iPS cell-derived primitive macrophage progenitors or iPS cell-derived primitive hematopoietic precursors can be transplanted into rodent brains^47,162–167^. Xenotransplantation protocols have been reviewed in more detail elsewhere^13,34,168^. For a successful transplantation and survival of the cells, immune deficiency and the expression of human colony stimulating factor 1 (CSF1) or interleukin (IL-34) are essential^162,169^. Xenotransplanted microglia remain functional and acquire typical microglia morphologies, as well as a transcription profile that more closely resembles ex vivo human microglia than cultured primary microglia^47,162,163,165–167^, indicating that many of the transcriptomic changes associated with 2D culture of iPS cells and human primary microglia can be corrected by xenotransplantation.

Furthermore, xenotransplantation enables the utilization of mechanisms used in MS animal models, such as cuprizone-induced demyelination^167^. Xu et al. were able to demonstrate that, in response to cuprizone-induced demyelination, CD74 and SPP1 expression in xenotransplanted microglia was upregulated, which is characteristic for disease-associated microglia^167,170–172^. These changes recapitulate observations made in patients with MS^173^.

While xenotransplantation of human microglia into rodent brains can restore some features of their in vivo identity, full acquisition of human-specific signatures and brain environment-dependent states remains lacking^47,162,163,165–167^. In addition, the prolonged survival of xenotransplanted human microglia is dependent on transgenic expression of human CSF1 or IL-34^162,169^. As a possibility to mitigate these shortcomings, Schäfer et al. engrafted immunocompetent human brain organoids^174^. Xenotransplanted brain organoids became vascularized and supported long-term survival of human microglia without providing exogenous human CSF1 or IL-34. The transcriptomic profile of organoid-resident human microglia was similar to that of their human in vivo counterparts, depicting a more mature adult-like homeostatic state and human brain environment-specific features. Moreover, human microglia displayed dynamic surveillance of their microenvironment and responded to local and systemic stimuli. Therefore, the model developed by Schäfer et al. offers the possibility to study human microglia in a human brain environment.

Although these xenotransplantation models create the opportunity to study and manipulate human microglia, closely resembling human brain microglia, in a mature CNS environment, and in the context of established MS animal models, high-throughput, large-scale studies remain very challenging due to the technical complexity of the models.

## Conclusion

The complexity of the mechanisms driving disease progression and the difficulty of obtaining primary human tissue make modeling MS progression particularly challenging. Furthermore, translating results from animal models to humans remains difficult due to species-to-species differences. Human iPS cell-derived technologies offer a promising solution to this challenge, with the potential to enhance our understanding of the pathophysiological mechanisms underlying MS progression and enable the development of effective drugs targeting disease progression.

The hugely successful development of protocols that enable differentiation of iPS cells into neurons, astrocytes, oligodendrocytes and microglia in both 2D and more complex 3D culture systems, such as spheroids or organoids, has become a very useful tool to study MS progression and provides new insights into its mechanisms (Fig. 1). Although none of these in vitro models can fully capture the complexity of MS, they show promising results in their ability to recapitulate certain mechanisms. Therefore, in vitro studies may substantially complement in vivo studies to overcome the species-to-species differences, and will help in the development of new therapeutic targets and treatment strategies for MS progression.
